# Ability of ^68^Ga‐PSMA PET/CT SUVmax to differentiate ISUP GG2 from GG3 in intermediate‐risk prostate cancer: A single‐center retrospective study of 147 patients

**DOI:** 10.1002/cam4.5516

**Published:** 2022-12-15

**Authors:** Nuo Yi, Yajing Wang, Shiming Zang, Lulu Yang, Hao Liu, Hongbin Sun, Liwei Wang, Feng Wang

**Affiliations:** ^1^ Department of Radiology, Nanjing First Hospital Nanjing Medical University Nanjing China; ^2^ Department of Nuclear Medicine, Nanjing First Hospital Nanjing Medical University Nanjing China; ^3^ Department of Pathology, Nanjing First Hospital Nanjing Medical University Nanjing China; ^4^ Department of Urology, Nanjing First Hospital Nanjing Medical University Nanjing China

**Keywords:** positron emission tomography, prostate cancer, prostate‐specific membrane antigen

## Abstract

**Objective:**

To evaluate the ability of ^68^Ga‐PSMA PET/CT maximum standard uptake value (SUVmax) to distinguish prostate cancer (PCa) International Society of Urological Pathology ISUP grade group (GG) 2 and GG3.

**Methods:**

The PET/CT images and data of 147 patients were analyzed retrospectively, and the SUVmax of the index lesions were measured. The receiver operating characteristic curve was used to analyze the diagnostic value of PET/CT for PCa. The correlation between SUVmax and ISUP GG was analyzed. A logistic regression model was established with SUVmax and was validated to predict its value of diagnosing intermediate‐ and high‐risk PCa (ihPCa).

**Results:**

Of the 147 patients, 112 cases were PCa (76.2%), and 35 cases were benign lesions (23.8%). There was a significant difference between the benign and the malignant groups (*p* < 0.05). The median SUVmax of ihPCa was significantly higher than that of the benign and low‐risk groups (*p* < 0.05). The median SUVmax of GG3 was significantly higher than that of the GG2 group (*p* < 0.05). There were no statistically significant SUVmax differences among GG3, GG4, and GG5 groups (*p* > 0.05). The specificity and the positive predictive value of ^68^Ga‐PSMA PET/CT in the diagnosis of PCa were 97% and 99% with cut‐off SUVmax of 6.94, while the specificity and the positive predictive value of ihPCa were 95% and 96% with cut‐off SUVmax of 10.12.

**Conclusion:**

^68^Ga‐PSMA PET/CT can reliably distinguish GG2 from GG3 PCa.

## Graphical abstract

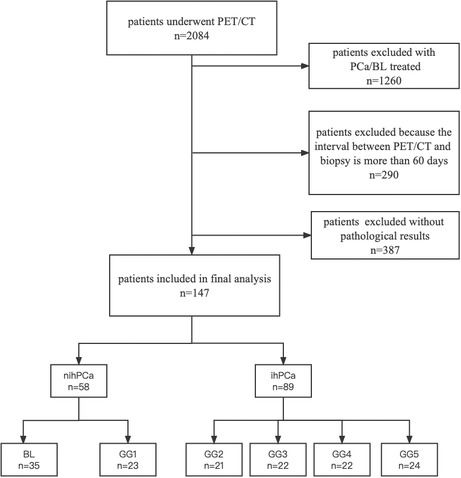

## 1 INTRODUCTION

Prostate cancer (PCa) is one of the most common cancers in elderly males worldwide, and the incidence of advanced PCa continues to increase.^1^ With the development of medical technology, the options of diagnosis and treatments for PCa are increasing gradually, but metastatic PCa is still an incurable cancer. Low‐risk PCa can be actively monitored to avoid surgery‐related complications such as incontinence, and intermediate‐ and high‐risk prostate cancer (ihPCa) can be treated with radical prostatectomy or endocrine therapy to improve the prognosis.^2^ Early diagnosis and treatment of PCa may improve the prognosis of the disease and the life quality of the patients. Serum prostate‐specific antigen (PSA) screening has been widely used in the general population, but the net benefits of PSA screening may be lower than the corresponding damage.^3^ Non‐invasive magnetic resonance imaging (MRI) can make accurate T‐staging of PCa, but it tends to miss some primary lesions and metastatic pelvic lymph nodes.^4^

As an inherent transmembrane protein existing in the prostate epithelial cell membrane, Prostate‐specific membrane antigen (PSMA) is highly expressed in more than 90% of PCa cells and increases with the augments in tumor invasion, metastasis, or recurrence.^5^, ^6^ Research in the last 6 years showed that ^68^Ga‐PSMA positron‐emission tomography/computed tomography (^68^Ga‐PSMA PET/CT) might provide higher diagnostic efficiency and more accurate staging and even detect the biochemical recurrence of PCa. Compared with MRI, ^68^Ga‐PSMA PET/CT can detect more recurrent and metastatic lesions.^7^, ^8^

^68^Ga‐PSMA PET/CT is a relatively new technique. The results of early diagnostic trials are very encouraging.^9^, ^10^ Because the sample sizes of many studies are not large enough, further verification through significant sample statistics is required. There already have articles that identify intermediate‐risk and high‐risk PCa, but there are few studies in identification GG2 and GG3. The prognosis of GG2 and GG3 is different, GG3 is more likely to metastasize and recur. It is meaningful to distinguish them in clinical management. The purpose of this study is to evaluate the ability of ^68^Ga‐PSMA PET/CT to distinguish prostate cancer (PCa) International Society of Urological Pathology ISUP grade group (GG) 2 and GG3. There is no relevant research literature up to now. Because this study has a relatively larger sample, we believe its results might be more credible and could provide practical guidance for nuclear medicine physicians and urologists.

## 2 MATERIALS AND METHODS

### 2.1 Clinical data

**FIGURE 1:**
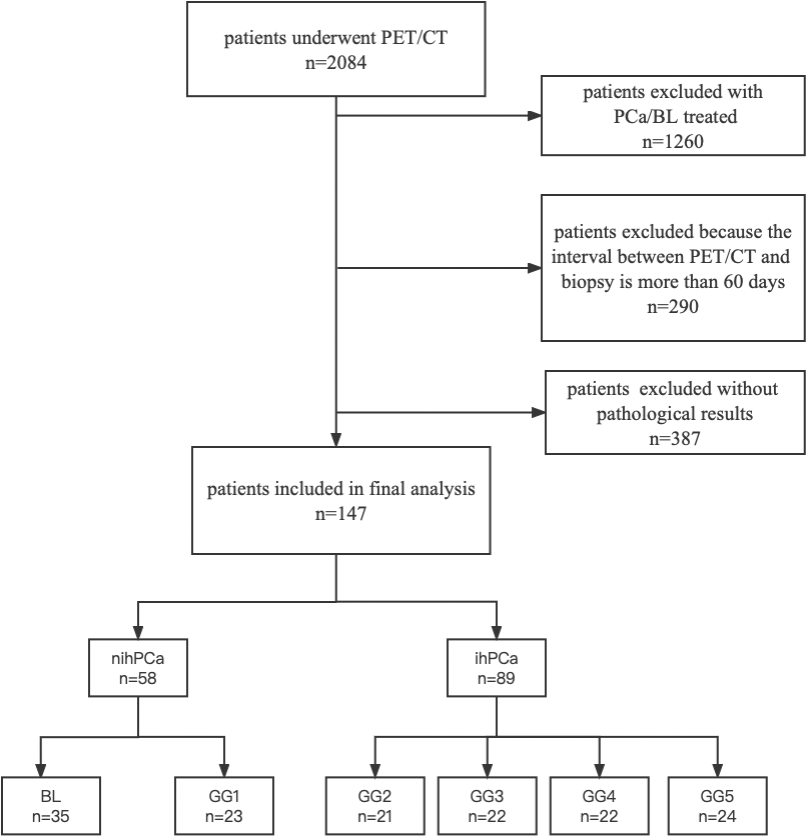
Flow chart of cases selection.

The data of patients who underwent PET/CT in our hospital from December 2016 to December 2021 were retrospectively analyzed. The patient's symptoms included dysuria, hematuria, and elevated PSA. Inclusion criteria for study cases: (1) The patients had suspicious PCa for the first time and underwent ^68^Ga‐PSMA PET/CT successfully; (2) The patients had not undergone endocrine or surgical treatment; (3) ^68^Ga‐PSMA PET/CT and needle biopsy performed within 30 days; (4) The exact pathological results were obtained. Exclusion criteria for study cases: (1) Patients had recurrent PCa; (2) The interval between PET/CT and needle biopsy was greater than 30 days; (3) Definite pathological results were not available. The flow chart of this study is shown in Figure 1. Finally, a total of 147 patients who met the criteria were enrolled in the study. The mean age of the patients was 71.7 ± 8.2 years (32–92 years). The PSA levels ranged from 1.23 to 2790 ng/ml. And 31 of the 147 patients underwent MRI. The hospital ethics committee approved this retrospective study.

### 2.2 PET/CT examination technique

^68^GA‐PSMA‐11 was synthesized using the automatic labeling module (ITG, Germany) in our institute. Our laboratory is the international four‐level standard (radiochemical purity >93%). ^68^Ga‐PSMA‐11 was given by intravenous injection, and the dose of injection was determined by body weight. The patients were instructed to drink more water and urinate more frequently. Spiral CT and PET/CT scanning were, respectively, performed 45 min and 60 min later (United Imagining, uMI780). The image acquisition range was from the top of the skull to the middle of the femur. PET acquisition took about 12 min with four‐bed positions. PET and CT were fused to obtain multi‐planar images manually.

### 2.3 MRI acquisition protocol

MRI was performed with a high‐field system (SIEMENS). The sequences included transverse T1‐weighted imaging (T1WI), T2‐weighted imaging (T2WI), fat‐suppression spectral presaturation attenuated inversion recovery‐T2WI, DWI and coronal T2WI. Scanning parameters: T1WI, repetition time (TR)/echo time (TE) = 441/10 ms; T2WI, TR/TE = 2800/105 ms; fat‐suppression spectral presaturation attenuated inversion recovery‐T2WI, TR/TE = 2800/100 ms; DWI, TR/TE = 4000/ 59 ms，b = 2000 s/mm^2^. The collection time is about 20 min. After the acquisition, the image will be transferred to the post‐processing workstation for analysis.

### 2.4 Image analysis

The PET/CT images were read with the double‐blind method. Two experienced nuclear medicine physicians analyzed the images, qualitatively diagnosed the index lesions, and staged the suspicious malignant lesions without knowing the clinical and pathological results. The two specialists measured the maximum standardized uptake value (SUVmax) of the index suspicious lesions of the prostate. Any disagreements of diagnosis between the two physicians were resolved through discussion.

The MRI images were reported by two experienced MRI radiologists using the standardized Prostate Imaging Reporting and Data System (PI‐RADS) version 2. Any disagreements of diagnosis between the two physicians were resolved through discussion. The PI‐RADS scores were classified as positive (PI‐RADS 4–5) or negative (PI‐RADS 1–3).

### 2.5 Pathological analysis

All patients underwent transrectal ultrasound‐guided prostate 12‐core needle biopsy (TUPB). The biopsy specimens were diagnosed and graded by pathologists. Benign lesions (BL) include normal prostate tissue, benign prostatic hyperplasia and prostatitis. Gleason score (GS) and International Society of Urological Pathology (ISUP) grade groups (GG) were both carried out. Pathological results were divided into five groups: GG1 (GS 6), GG2 (GS 3 + 4 = 7), GG3 (GS 4 + 3 = 7), GG4 (GS 8), and GG5 (GS 9–10). GG1 was defined as low‐risk PCa, GG2‐3 were intermediate‐risk PCa, and GG 4–5 lesions were high‐risk PCa (hPCa).^11^, ^12^ GG 2–5 were intermediate‐ and high‐risk PCa (ihPCa). BL and low‐risk PCa were non‐ihPCa (nihPCa).

### 2.6 Statistical analysis

Quantitative data conforming to normal distribution are recorded as mean ± standard deviation and compared with the independent‐sample *t*‐test. The data that do not conform to the normal distribution are expressed as median [25th, 75th percentile] and compared with the Mann–Whitney U test. Spearman correlation analysis was carried out to determine the correlation between SUVmax, ISUP GG and PSA levels. The differences of SUVmax between the benign group and the malignant group were compared. The data were analyzed with IBM SPSS version 26.0 (IBM, Inc.) and GraphPad Prism version 9.0 (GraphPad Software, Inc.). The receiver operating characteristic curve (ROC) of PET/CT and PI‐RADS were drawn, and the area under the curve (AUC) were calculated. Differences were statistically significant when *p* < 0.05.

## 3 RESULTS

**FIGURE 2:**
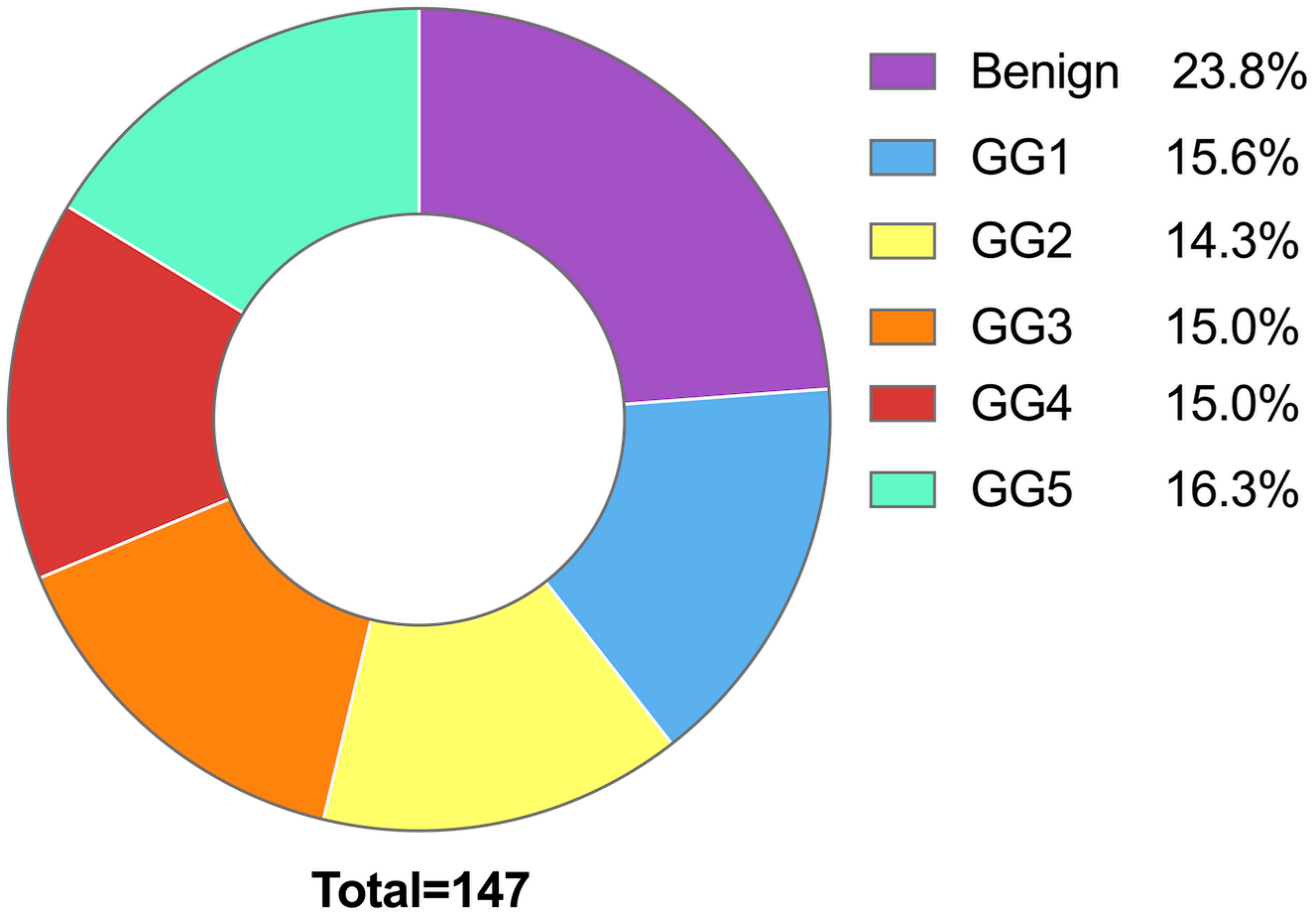
Distribution of pathological results of all cases.

**FIGURE 3:**
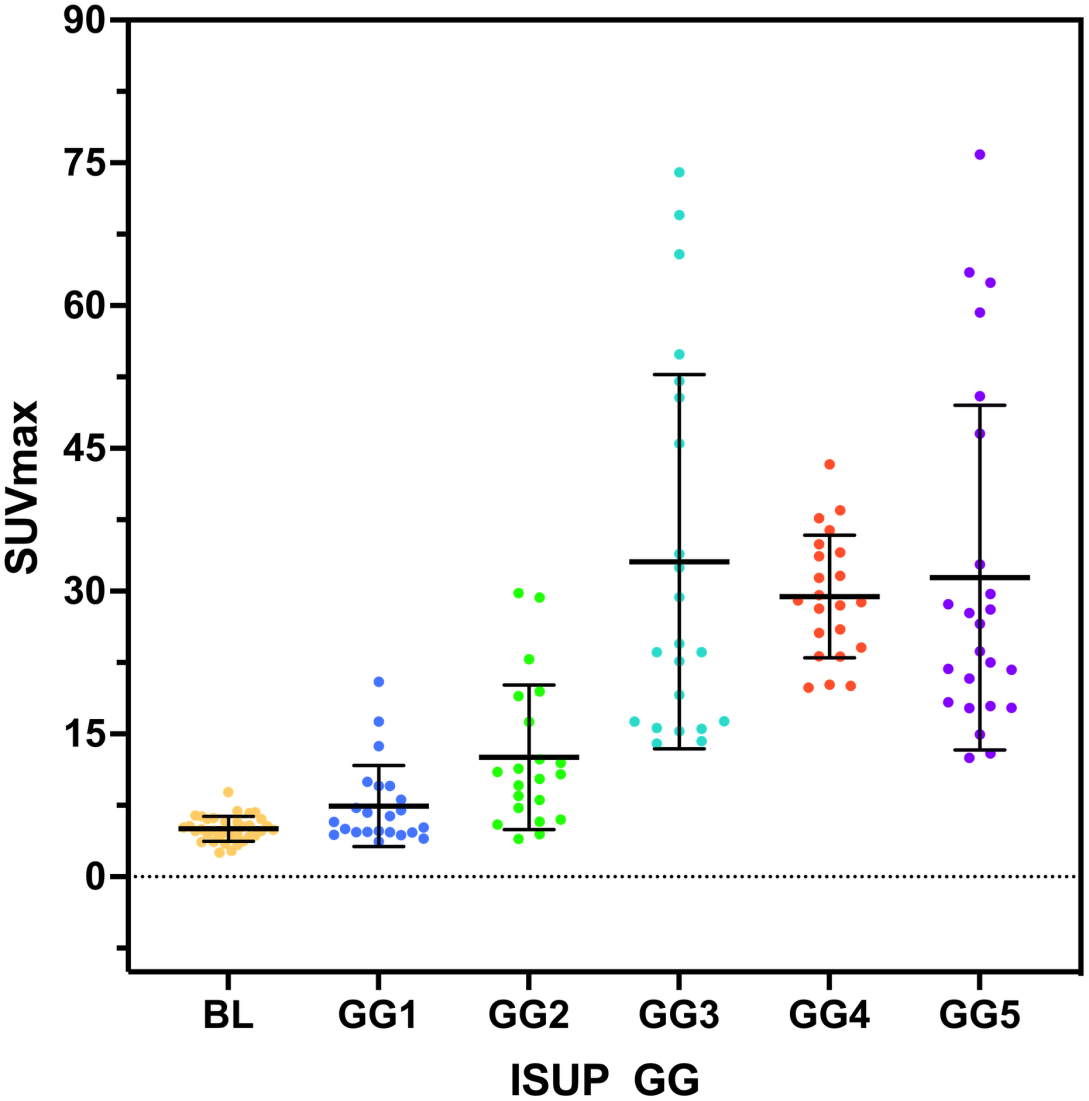
Scatter dot plot of the SUVmax distribution of all cases.

**FIGURE 4:**
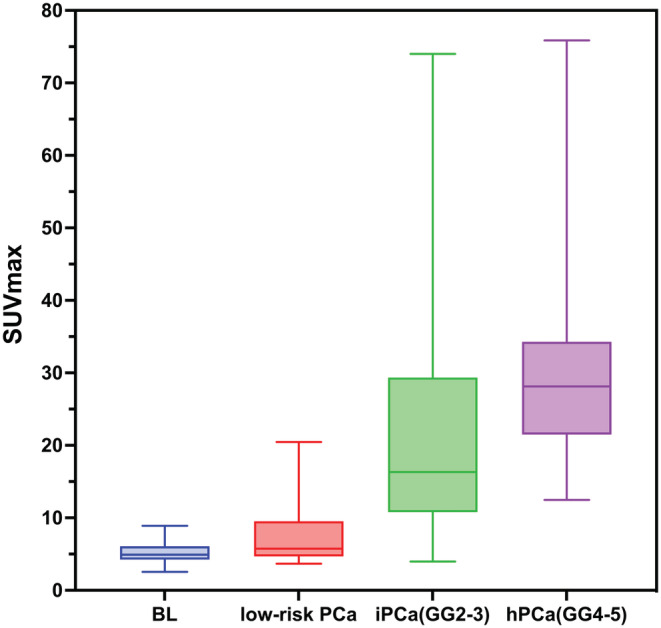
Box plot of the SUVmax distribution of different risk groups.

**FIGURE 5:**
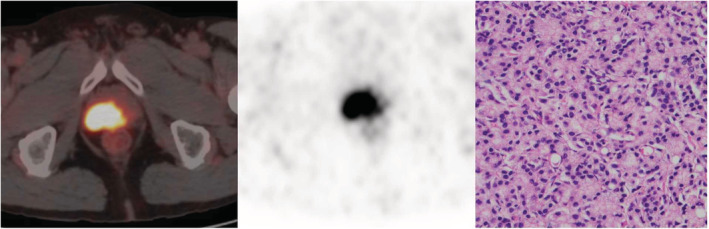
A 73‐year‐old patient with progressive dysuria for 5 years (SUVmax = 12.94, PSA: 58.67 ng/ml). (A, B) Obvious radioactive uptake was showed on PET/CT (white arrow), (C) prostate cancer (grade group 5) was proven by pathology (hematoxylin and eosin staining, 100× magnification).

**FIGURE 6:**
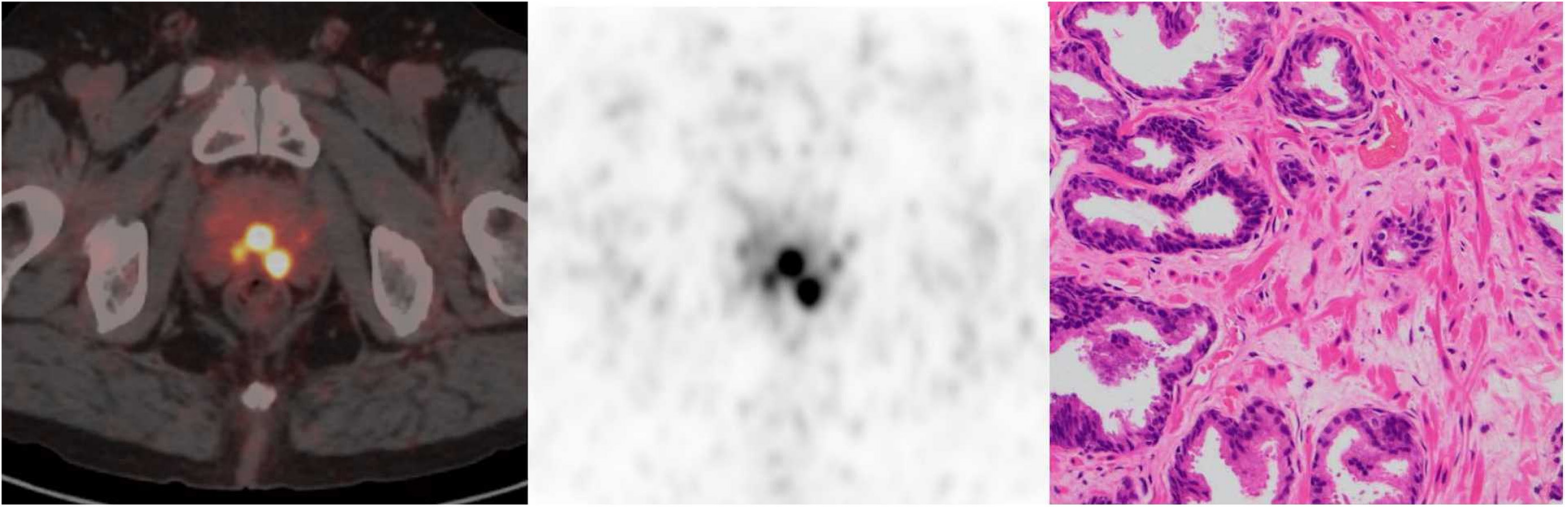
A 70‐year‐old patient with progressive dysuria for 5 years (SUVmax = 11.11, PSA: 10.00 ng/ml). (A, B) Radioactive uptake was increased on PET/CT (white arrow), (C) pathology results was benign prostate tissue (hematoxylin and eosin staining, 100× magnification).

One hundred and forty seven patients underwent ^68^Ga‐PSMA PET/CT and biopsy totally. Among them, 112 (76.2%) had PCa (ISUP GG1‐5), and 35 (23.8%) had benign lesions (see Figure 2). The most common ISUP GG were GG5 (*n* = 24, 16.3%) followed by GG1 (*n* = 23, 15.6%) and GG3/4 (*n* = 22, 15.0%). The data of median SUVmax among groups were displayed in Table 1. The radioactive concentration of BL is shallow, similar to or slightly higher than normal tissue, and a few have moderate or higher radioactive concentrations. PCa images have higher radioactive concentrations than normal tissues. The SUVmax distribution of different risk groups is shown in Figures 3 and 4. The images of typical cases are shown in Figures 5 and 6.

**TABLE 1:** Distribution of median of SUVmax

Groups	Cases (*n* = 147)	SUVmax Median (P25, P75)
BL	35	4.91 (4.25,5.92)
nihPCa	58	5.11 (4.43,6.42)
Malignant	112	19.95 (9.88,29.41)
ihPCa	89	23.59 (15.63,32.47)
GG1	23	5.74 (4.69,8.81)
GG2	21	10.78 (7.24,16.27)
GG3	22	24.03 (16.32,49.12)
GG4	22	28.93 (24.47,33.96)
GG5	24	25.12 (18.23,36.25)

There was a positive correlation between SUVmax and PSA levels (*ρ* = 0.54, *p* < 0.001). The median PSA level of GG1 was higher than that of BL, but there was no significant difference between the two groups (*p >* 0.05). The median SUVmax of GG1 was 5.74, which is higher than 4.91 of BL, and the difference was significant (*p* < 0.05).

There was a positive correlation between SUVmax and ISUP GG (*ρ* = 0.68, *p* < 0.001). The median SUVmax of ihPCa was significantly higher than that of BL, the low‐risk, and the nihPCa groups (*p* < 0.05). The median SUVmax of GG3 was higher than that of the GG2 group, and the difference was significant (*p* < 0.05). There were no statistically significant SUVmax differences among GG3, GG4, and GG5 groups (*p* > 0.05). In the diagnosis of PCa, the cut‐off value of 6.94 achieved a specificity of 97% and a positive predictive value (PPV) of 99%. The specificity and the PPV of ihPCa were 95% and 96%, respectively, with the cut‐off value of 10.12. After drawing the ROCs, the diagnostic parameters of PCa, ihPCa, and high‐risk PCa were calculated, and the results were shown in Table 2. After classifying GG3 as high‐risk PCa, the sensitivity, specificity, PPV, negative predictive value (NPV), accuracy, Youden index and AUC were 100%, 89%, 88%, 100%, 94%, 0.89 and 0.97, respectively.

**TABLE 2:** The results of PCa with ^68^Ga‐PSMA PET/CT

Groups	Cut‐off (SUV_max_)	Sensitivity	Specificity	Youden index	AUC	PPV	NPV	π
PCa (all)	6.94	0.83	0.97	0.80	0.91	0.99	0.64	0.86
ihPCa (≥GG 2)	10.12	0.90	0.95	0.85	0.96	0.96	0.86	0.92
hPCa (≥GG 4)	17.03	0.93	0.79	0.72	0.87	0.67	0.96	0.84
PCa (≥GG 3)	12.41	1.00	0.89	0.89	0.97	0.88	1.00	0.94

**FIGURE 7:**
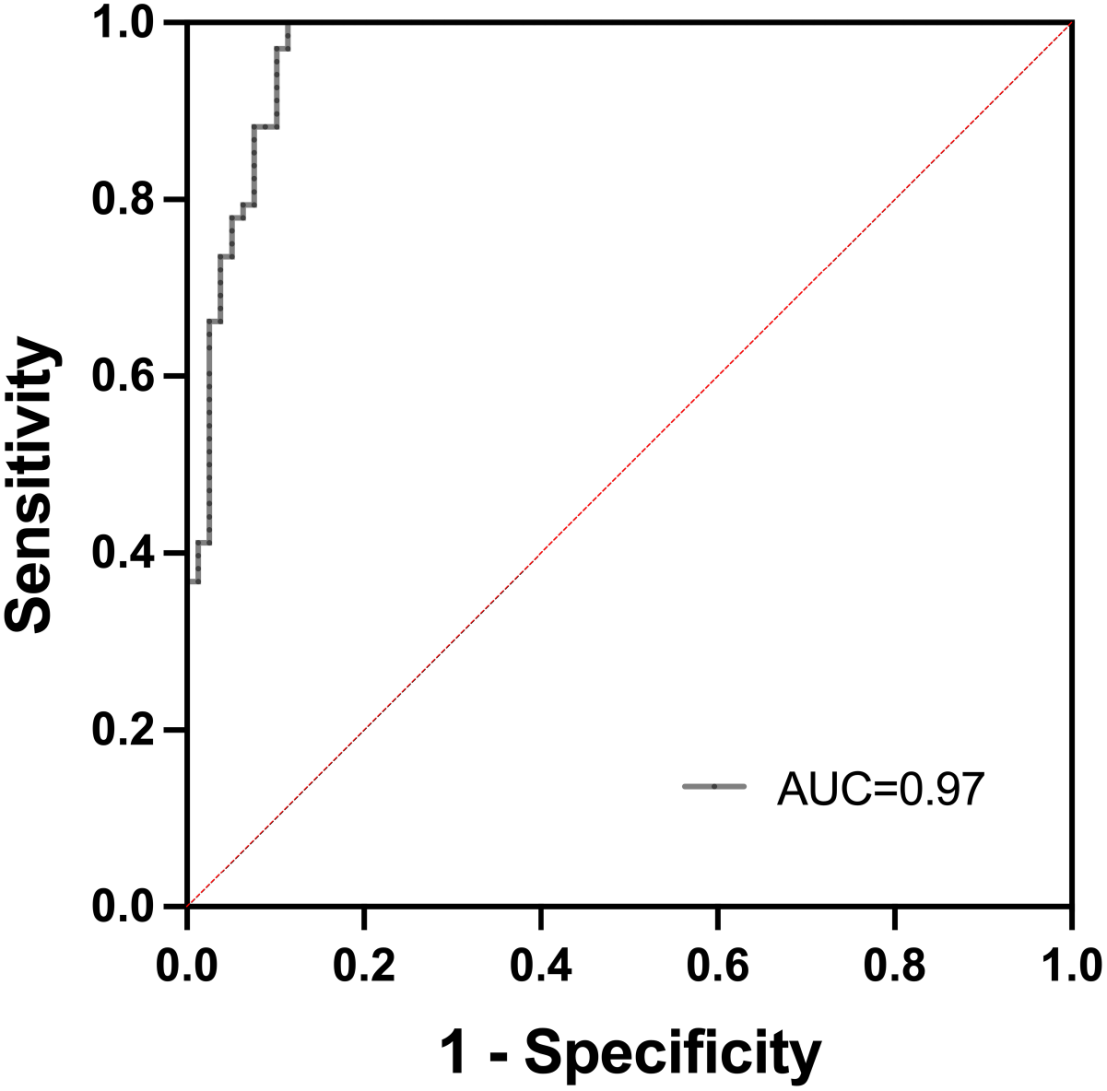
ROC of the regression model for predicting PCa (≥GG3).

To further evaluate the predicting ability of ^68^Ga‐PSMA PET/CT, SUVmax was entered as independent variables of a logistics regression, and the patients' conditions (benign, GG1‐2 and GG≥3) were entered as binary outcome variables. The regression model for predicting PCa(GG≥3) was established based on the SUVmax of 147 patients: logit (p) = −5.048 + 0.3129 × SUVmax. The results showed that the SUVmax was an independent predictor for PCa(GG≥3), and Youden index was 0.6, with a sensitivity of 88% and specificity of 90%. The ROC for predicting PCa(GG≥3) is shown in Figure 7.

In the diagnosis of PCa, the sensitivity, specificity, PPV, NPV and AUC of MRI (PI‐RADS 4–5) were 68%, 89%, 94%, 53% and 0.77, respectively. The sensitivity, specificity, PPV, NPV and AUC of ihPCa were 82%, 44%, 78%, 50% and 0.72, respectively.

## 4 DISCUSSION

Early detection is the key to improve the survival rate of PCa patients. Correct grading of PCa helps choose the appropriate treatment plan. This study showed that ^68^Ga‐PSMA PET/CT had good performance in the diagnosis of PCa. The ISUP GG and PSA levels correlated with the SUVmax of patients with PCa on ^68^Ga‐PSMA PET/CT. It not only can distinguish the benign lesions from the malignant but also can differentiate between ISUP GG2 and GG3 PCa. Thus, it can be used to distinguish G2 or higher overall. This research can improve the diagnostic confidence of nuclear medicine physicians and help urologists understand this examination method further.

PSA measurement is the first choice for screening PCa, but there was no significant difference between GG1 and BL groups, while the SUVmax had a significant difference between the two groups. The PSMA expression in patients with GG1 PCa was higher than that in patients with BL lesions, which can be used to distinguish between benign prostate tissue and GG1. TRUB(transrectal ultrasound‐guided biopsy) is a minimally invasive examination, but it has complications such as bleeding or infection, and some patients may miss the best treatment chance because they reject TRUB.^13^ Among non‐invasive imaging methods, multi‐parameter MRI has higher sensitivity in diagnosing prostate cancer than ultrasound or CT. However, it still has false positives and false negatives.^14^, ^15^ According to Kitajima et al.^16^ the sensitivity of PET/CT with 18F‐FDG as the probe in diagnosing primary PCa is only 45.5%. At present, targeted PSMA PET/CT is considered as the most promising method for PCa staging and management. Since the European Association of Urology recommended PSMA PET/CT in 2017, its application in PCa has been widely promoted.^17^ For patients who are suspicious of PCa with high PSA levels and suspicious lesions and refuse to do a needle biopsy, PET/CT provides them with the opportunity for early detection and early treatment. However, some of them may prove to be benign lesions. Considering cost‐effectiveness and radioprotection issues, it should be cautious when PET/CT is recommended.

The grading of PCa is related to its prognosis, and urologists will choose the best‐individualized treatment plan according to the risk stratification of PCa.^18^ Predicting the risk stratification of PCa correctly before the operation is helpful to improve the patients' and physicians' understanding of the disease conditions. Current research shows that PSMA uptake concentration is closely related to GS.^19^ Woythal et al. showed that ^68^Ga‐PSMA PET/CT had a significantly higher SUVmax in PCa than normal prostate tissue.^20^ The SUVmax was significantly correlated with the expression of PSMA in the primary tumor. With 3.15 as the cut‐off value of SUVmax, the sensitivity, specificity, and AUC of ^68^Ga‐PSMA PET/CT in detecting PCa were 97%, 90%, and 0.987, respectively. The SUVmax cut‐off value of this study was 6.94, which was higher than that of Woythal et al.^20^ but was similar to that mentioned in the last 2 years.^21^ The reasons for the significant difference in the cut‐off values may include sampling errors, different equipments, or different injection doses.

Demirci et al. retrospectively evaluated 141 patients who underwent ^68^Ga‐PSMA PET/CT imaging and prostatectomy.^21^ The results showed that the mean SUVmax of GG3 PCa (13.3 ± 8.5) was significantly higher than that of GG2 PCa (7.4 ± 4.6). The mean SUVmax of patients with high‐risk PCa (GG3‐5) (18.9 ± 12.1) was significantly higher than that of patients with low‐risk PCa (GG1‐2) (7.16 ± 6.2). ROC analysis (AUC = 0.85) revealed that the sensitivity and specificity of PCa diagnosis were 78% and 81%, respectively, when 9.1 was taken as the cut‐off value. In this study, the diagnostic performance of ^68^Ga‐PSMA PET/CT for ISUP grading of PCa was analyzed, and this study classified GG3 PCa as intermediate‐risk PCa. The results obtained were similar to those of Demirci et al.^21^ but the SUVmax of ihPCa in our study was generally higher than theirs. This may be related to different machine types. In our research, the larger the SUVmax of PCa, the higher the ISUP GG of PCa. These two have a relatively strong positive correlation. Our study shows that the median SUVmax of ihPCa was significantly higher than that of nihPCa, which indicated that ^68^Ga‐PSMA PET/CT had better performance in detecting clinically significant PCa. The difference was significant (*p* < 0.05). SUVmax could predict ihPCa, which could guide the formulation and adjustment of patients' treatment plans. In our study, when GG3 lesions were classified as high‐risk PCa, ROC analysis showed that the Youden index, accuracy, and AUC value of SUVmax in diagnosing high‐risk PCa were significantly higher than the original classification method. That is, GG3 lesions should be classified as high‐risk PCa. This may be related to the fact that the median SUVmax of GG3 lesions was closer to that of GG4‐5 lesions and significantly higher than GG2 lesions. When GG3 lesions are classified as intermediate‐risk PCa, there are more false‐positive cases when calculating high‐risk PCa, which could reduce diagnostic specificity and positive predictive value. Therefore, classifying GG3 lesions into high‐risk PCa would have higher diagnostic accuracy in this study. Since GG3 is currently classified as intermediate‐risk PCa in most literature, the same classification was also done in this study. However, in this study, SUVmax of GG3 PCa was higher than GG2 PCa, which was almost equal to that of GG4‐5, which indirectly verified the high risk of GG3 PCa and GG3 should be classified as high‐risk PCa. ^68^Ga‐PSMA PET/CT provides indirect evidence that GG3 PCa is riskier than GG2.^22^, ^23^, ^24^ The former should be given more radical treatment, so it is recommended to classify GG3 PCa as a high‐risk PCa in subsequent clinical practice and research.

In this study, the SUVmax of GG1 PCa was significantly lower than that of ihPCa, and there were significant differences from the SUVmax of benign lesions. Therefore, ^68^Ga‐PSMA PET/CT could distinguish reliably low‐risk PCa from benign lesions. Different results are reported in the literature. Scheltema et al. found that the sensitivity of ^68^Ga‐PSMA PET/CT in diagnosing GG1 lesions was very low (18%).^25^ This result means that ^68^Ga‐PSMA PET/CT might miss most part of the GG1 PCa. However, the number of patients included in this study was only 56, which was far lower than the number of 147 patients in our study, so we believe that the results obtained in our study based on larger sample size are more accurate.

Based on the MRI scores of 31 patients who underwent MRI, it can be found that MRI has relatively high specificity and PPV in diagnosing PCa, but the specificity and PPV in diagnosing ihPCa are both low. It has proved that the PI‐RADS score is of little value in the diagnosis of ihPCa in this study. In addition, compared with the specific value of SUVmax, the PI‐RADS score is relatively subjective, which will also affect the accuracy of diagnosis.

PET/CT provides a more accurate non‐invasive imaging method for patients who are suspicious of PCa. Moreover, because of its high specificity and positive predictive value in diagnosing PCa and ihPCa, PET/CT also has a certain prospect in the non‐invasive diagnosis of PCa. The precise guidance of PET/CT can help the urologists to know better about the location of the tumor and the relationship between the tumor and the surrounding tissues, determining the safe range of surgical resection and making the operation more accurate, safe, and effective.

### 4.1 Limitations

The limitation of this study is that we used a biopsy to obtain all the pathological results, and there may be a matching error between the puncture sites and PET/CT lesions. It is recommended that the whole specimen after radical prostatectomy be compared with PET/CT in future research to avoid deviation.

## 5 CONCLUSIONS

^68^Ga‐PSMA PET/CT has good performance in the diagnosis of prostate cancer. It not only can distinguish the benign lesions from the malignant but also can differentiate between ISUP GG2 and GG3 PCa. ^68^Ga‐PSMA PET/CT SUVmax showed high specificity and positive predictive value in diagnosing PCa and ihPCa. ISUP GG3 PCa should be classified as high‐risk PCa. This research can improve the diagnostic confidence of nuclear medicine physicians and help urologists understand this examination method further.

## AUTHOR CONTRIBUTIONS

**NUO YI:** Conceptualization (lead); data curation (lead); formal analysis (lead); investigation (equal); writing – original draft (lead); writing – review and editing (equal). **Yajing Wang:** Conceptualization (equal); data curation (supporting); formal analysis (supporting); writing – review and editing (equal). **Shiming Zang:** Formal analysis (supporting); resources (supporting); writing – review and editing (supporting). **Lulu Yang:** Data curation (supporting); supervision (supporting); validation (supporting). **Hao Liu:** Data curation (supporting); supervision (supporting); validation (supporting). **Hongbin Sun:** Data curation (supporting); supervision (supporting). **Liwei Wang:** Conceptualization (equal); formal analysis (supporting); investigation (supporting); supervision (equal); validation (supporting); visualization (equal); writing – original draft (supporting); writing – review and editing (supporting). **Feng Wang:** Data curation (supporting); investigation (supporting); supervision (equal); visualization (supporting); writing – review and editing (supporting).

## FUNDING INFORMATION

This research was supported by grants from the Special funds for key research and development plan (social development) of Jiangsu Province (BE2021605).

## CONFLICT OF INTEREST

The authors declare that they have no conflict of interest.

## ETHICS STATEMENT

All procedures performed in this study involving human participants were carried out in accordance with the ethical standards of the Nanjing First Hospital and/or National Research Committee.

## Data Availability

Not applicable.
